# An integrated intervention of computerized cognitive training and physical exercise in virtual reality for people with Alzheimer's disease: The jDome study protocol

**DOI:** 10.3389/fneur.2022.964454

**Published:** 2022-08-12

**Authors:** Elena Gambella, Arianna Margaritini, Marco Benadduci, Lorena Rossi, Paola D'Ascoli, Giovanni R. Riccardi, Sara Pasquini, Patrizia Civerchia, Giuseppe Pelliccioni, Roberta Bevilacqua, Elvira Maranesi

**Affiliations:** ^1^Neurology Unit, IRCCS INRCA, Ancona, Italy; ^2^Scientific Direction, IRCCS INRCA, Ancona, Italy; ^3^Clinical Unit of Physical Rehabilitation, IRCCS INRCA, Ancona, Italy

**Keywords:** Alzheimer's disease, virtual reality, cognitive rehabilitation, physical rehabilitation, study protocol

## Abstract

**Introduction:**

Alzheimer's disease is a neurodegenerative syndrome characterized by cognitive deficits, loss of daily functions, and mental and behavioral disorders, which cause stress and negatively affect the quality of life. Studies in the field suggest that combining cognitive training with physical activity can reduce the risk of developing the disease and, once neurodegeneration has begun, it slows its progress. In particular, virtual reality and augmented reality administer cognitive stimulation while providing a link to autobiographical memory through reminiscence, enabling the improvement of the person's quality of life. The present protocol aims to evaluate the effectiveness of cognitive and physical treatments, integrated with the addition of virtual reality and reminiscence elements, using the Brainer software, in which people will find cognitive training, and the jDome^®^ BikeAround™ system, which will allow participants to pedal along a personalized path projected on a schematic, using an exercise bike connected to the system.

**Methods and analysis:**

For this study, 78 patients with mild Alzheimer's dementia were recruited and divided into the Experimental Group (EG) and Control Group (CG). Sixteen treatment sessions of 60 min each were conducted for both groups (2 training sessions per week, for 8 weeks), including 1 patient at a time. The EG received cognitive treatment with Brainer and physical training with jDome, while the CG received cognitive treatment with Brainer and physical training with a classic bicycle. The evaluation mainly focused on the assessment of the person's cognitive status. Other analyses were conducted on the quality of life, mood, behavioral disorders, and physical function, which were considered secondary outcomes.

**Discussions:**

The ultimate goal of the present study is to test the effectiveness of a treatment for people with mild Alzheimer's focused on the integration of cognitive training and aerobic physical activity, using an exercise bike, with the addition of virtual reality and reminiscence elements.

**Ethics and dissemination:**

The study was approved by the Ethics Committee of the IRCCS INRCA. It was recorded in ClinicalTrials.gov on 2 June 2022 with the number NCT05402423. The study findings will be used for publication in peer-reviewed scientific journals and presentations in scientific meetings.

## Introduction

Dementia is a chronic neurodegenerative syndrome characterized by deficits in cognitive functions, associated with the loss of daily function and with mental and behavioral disorders. There are over one million people with dementia in Italy, of which 54% are due to Alzheimer's disease and about 16% are due to vascular dementia (1). Alzheimer's disease is a neurodegenerative disease with insidious onset and progressive impairment of behavioral and cognitive functions, including memory, comprehension, language, attention, reasoning, and judgment (2, 3). Neuropsychiatric symptoms, like apathy, social withdrawal, disinhibition, agitation, psychosis, and wandering, are sources of stress and negatively affect the quality of life of both the person with dementia and their caregiver (4).

There is no cure for Alzheimer's disease, although psychosocial interventions have been shown to be effective in improving the quality of life and psychological wellbeing of people with dementia (5–8); moreover, they are considered the treatment of choice for the management of psychological and behavioral disorders (9, 10).

Psychosocial interventions include a wide variety of strategies, ranging from emotional and behavioral approaches, physical training to cognitive stimulation, and psychological therapy (11). Psychosocial interventions aim to maximize the use of the remaining function, recruiting a compensatory network and preventing the disuse of brain function acting on the cognitive reserve, neural plasticity, and residual abilities of the person with dementia (12). Neural plasticity refers to the ability of the brain to change itself based on experiences that have been lived and learning throughout the life span. This ability is also maintained in Alzheimer's patients by determining modifications and the growth of some neuronal and synaptic pathways in the central nervous system, such as in the hippocampus and the entorhinal cortex. Stimulating neuroplasticity helps the person to compensate as much as possible for the damage produced by the disease. Moreover, also in the early/moderate stages of AD, cognitive reserve, which helps in the efficient use of strategies and functional connection networks between pre-existing neurons, compensates for neurodegeneration and allows the maintenance of patients' cognitive performance (13). Psychosocial interventions, therefore, do not alter disease progression in terms of structural neuropathology, but they act through functional brain changes and alter the structure of the association networks, like the default mode network connectivity (14, 15).

According to literature, cognitive interventions (16, 17), regular physical activity (9, 10), and reminiscence therapy appeared effective in the therapy of people with mild to moderate dementia (17).

Cognitive stimulation programs, computerized individual cognitive training, as well as physical exercise appear to be able to induce a significant improvement in cognitive performance, quality of life, and wellbeing in people with mild to moderate dementia (18–20).

Physical activity increases the availability of different classes of growth factors that stimulate hippocampal neurogenesis (21), neuronal plasticity (22), and the reduction of symptoms of depression (23). Several studies show, especially in the early stages of the disease, that stimulation and participation in various types of activities help to counterbalance the cognitive changes related to the pathology, thanks to cognitive plasticity (24, 25). Although in a recent review it emerged that the results of studies on the subject are inconsistent and of poor quality (26), other studies suggest that physical activity is able not only to delay the onset and reduce the risk of Alzheimer's disease but also to slow the functional decline after the onset of neurodegeneration (23, 27).

People with Alzheimer's disease who practice physical activity reported an improvement in daily function, a slowing of the decline in cognitive tests, a better physical performance associated with a reduced risk of falling, and a decrease in depressive symptoms compared to those who do not practice physical activity (23, 28). The WHO guidelines on reducing the risk of cognitive decline and dementia suggest that healthy elderly people perform at least 150 min of moderate-intensity aerobic physical activity per week (29). However, it has not yet been identified which type of systematic and structured physical activity is most effective in reducing the risk or slowing down cognitive decline in the elderly (30). There are currently no specific guidelines on what type of exercise (aerobic, strength training, balance training, or a combination of all of these), how often, and how long is most beneficial for specific forms, and the severity levels of dementia. (31). At the moment, multicomponent physical exercise is the most effective, bringing improvements in daily functionality and promoting autonomy (30). However, recent studies underline the need to take into account the risk of adverse events both physically (e.g., increased risk of falling, cardiovascular events during exercise) and the cognitive deterioration for people with dementia who adhere to aerobic exercise programs and of resistance, especially if of high intensity (31, 32).

Studies suggest that combining physical exercise with cognitive training may be a more successful strategy for promoting greater stability of cognition, improvement in apathy, mood, autonomy in daily living activities, and quality of life compared to physical exercise alone (33–35).

According to the literature, the exercise bike appears to be suitable for carrying out aerobic physical activity for people with dementia, and virtual cycling is an even more engaging and stimulating alternative to do it (36–38). This type of cycling makes it possible to recall the pedaling of youth and seems to encourage higher levels of physical activity (38). Furthermore, virtual reality and augmented reality offer the possibility of simultaneously providing cognitive stimulation and a connection to autobiographical memory through reminiscence, thus participating in the improvement of the quality of life of people with dementia (39). The World Health Organization stressed the importance to use technology to improve the treatment of dementia (40). Virtual experiences improve mood and reduce apathy and allow people with dementia to be simultaneously engaged in meaningful conversations, as well as in exercise activities (39, 41). In fact, virtual experiences are generally preferred by people with cognitive impairment over non-virtual experiences (39).

Karssemeijer et al. demonstrated that both interactively combined physical exercise with cognitive training in a virtual environment and standard aerobic training can induce an improvement in psychomotor speed, an important predictor of functional decline (33).

Finally, virtual reality can also be used to stimulate reminiscence and the recovery of more vivid memories in people with Alzheimer's (42). Reminiscence is a non-specific stimulation activity that consists of recalling in a structured way personal past events supported by memory triggers. The recall of memories from autobiographical memory (e.g., significant events in the person's life) becomes the tool to favor the recovery of emotionally pleasant experiences. Reminiscence therapy produces an improvement in the quality of life and mood of people with dementia (42–48).

Following this review of the literature and taking into account the limits already identified by the authors of the studies discussed above, we want to evaluate the effectiveness of an integrated cognitive and physical treatment with added virtual reality and reminiscence elements.

The training will be administered in an individualized way and will consist of two separate components carried out in sequence: first, a computerized cognitive training with the web platform “Brainer—Professional Brain trainer” (49) *via* tablet, and then a physical training motivated by reminiscence through the jDome^®^ BikeAround™ (in the text named jDome) system (50, 51), which connects a stationary bicycle to a monitor. The Brainer Professional web platform is a clinically proven medical device for the cognitive rehabilitation of people with mild-stage dementia (49).

The cognitive exercises on the Brainer platform aim to help participants use compensatory strategies and exploit their residual cognitive skills to improve or maintain daily functions. Therefore, the Brainer platform aim at modify the course of the disease, allowing participants to maintain their autonomy longer and to reduce the lack of interest, anxiety and depression that dementia entails.

The use of the jDome system, on the other hand, will allow participants to cycle through an individualized path projected digitally on a screen. The support of a psychologist will allow participants to recover memories and emotions associated with the places encountered along the way in a reminiscence activity. jDome offers the participant the opportunity to engage in a motivating reminiscence activity associated with physical activity, this association can have a positive impact on maintaining physical and cognitive abilities and reducing the risk of falling (50, 51). According to the literature, exposure to an environment rich in stimuli associated with the specific past life of each participant allows for an improvement in the autobiographical memory and the quality of life (44). It can be assumed that the possibility of virtually retracing the streets and places of one's youth also allows the person using jDome to achieve a similar result.

The jDome is a tool developed in Sweden that takes people with dementia on a virtual bicycle tour along the paths of memories (51). The jDOME device combines an exercise bike, a dome-shaped projector, and Google Street View technology. The destination is typed into the computer, and once the image is loaded it appears on the screen. Users sit on the bike and cycle to meaningful destinations (a childhood home, a vacation destination, the place where they got married) projected onto the screen in front of them. The set of pedals and bicycle handlebars placed in front of the screen allows people to move the image and control what they are seeing around them, as well as giving the person the feeling of actually setting the movement. Moreover, the type of activity proposed by the jDome system stimulates not only physical performance but also implicitly the visuospatial skills and spatial orientation of the subject. The system is an innovative program that encourages physical and cognitive training in a fun and interactive way (50). For the purposes of the study, an individualized path will be structured on the basis of the roads and places significant for each participant from which to recover life episodes and reactivate the emotions linked to them in the hic et nunc. Selecting the places where the person can virtually pedal allows to prepare an individualized multisensory multimedia environment to make the most of the potential of technology in the therapy of reminiscence (44).

There is currently a lack of studies on the effectiveness and usefulness of jDome system. One study reported a limited benefit in mental health and wellbeing for people with dementia who used the system in residential settings (52).

From the interviews, it is clear that the greatest benefits derived from using jDome fall within the emotional sphere because the mood improves by bringing to mind many good times past. Most people were happy after using jDome, while only a few had negative experiences of sadness and anxiety (52).

For the purpose of studying the effectiveness of a treatment based on the association of computerized cognitive training that uses the “Brainer” web platform *via* tablet to a virtual reality system for aerobic physical exercise (jDome), changes will be assessed in cognitive level, physical performance, and quality of life of people with mild Alzheimer's dementia.

The study we are going to describe is within the InnFamiglia Project, funded by the Cariverona foundation as part of the Welfare & Family calls. The consortium is coordinated by the INRCA (Ref. Code 2017.0143).

## Methods and analysis

### Trial design

The study is designed as a single-blinded (outcome assessors) randomized controlled trial to test the effectiveness of a training program that combines the jDome virtual reality system for aerobic exercise with computerized cognitive training *via* tablet. The Experimental Group (EG) will receive computerized cognitive training with the Brainer web platform and aerobic training with the jDome system. The Control Group (CG), on the other hand, will receive computerized cognitive training with the Brainer web platform and aerobic training with a standard exercise bike.

Assessment will be performed at the baseline (T0), at the end of intervention (after 8 weeks-T1), and 3 months after the end of intervention (follow-up T2).

The primary aim is to assess the stabilization of the global cognition of people with mild-stage Alzheimer's disease at the Mini-Mental State Examination (MMSE) and at the Alzheimer's Disease Assessment Scale-Cognitive Subscale (ADAS-Cog).

The secondary aim is the analysis of the modification of the quality of life, mood, behavioral disturbances, and physical function in people with mild-stage Alzheimer's disease.

### Study setting

The study will be conducted at the Center for Cognitive Disorders and Dementia (CDCD) of the Neurology Unit of the Istituto Nazionale Ricovero e Cura per Anziani IRCCS INRCA, Ancona, Italy. The last version (second version) of the current protocol is dated 8 March 2022.

### Participants

The inclusion criteria are:

- Aged 65 and over;
- Pre-existing diagnosis of Alzheimer's, in mild phase, according to the 2011 criteria of the National Institute on Aging-Alzheimer's Association (NIA-AA);
- Clinical Dementia Rating Scale CDR = 1;
- Mini-Mental State Examination MMSE > 19;
- Functional Ambulation Categories FAC ≥4;
- Tinetti scale ≥ 20;
- Presence of a contact family caregiver
- Residing at home.

The exclusion criteria are:

- Failure to meet the inclusion criteria
- Sensory deficits not compensated by the use of prostheses
- Psychological and behavioral disorders not compensated by drug treatment
- Medical contraindication to moderate intensity aerobic exercise.

### Sample size

The sample size estimation for this study was carried out considering the changes in MMSE score and ADAS for the cases compared to controls as the main outcomes. In the study by Han et al. (53), 64 subjects with MCI or mild dementia were enrolled to take part in combined cognitive training, cognitive stimulation, reality orientation, physical therapy, reminiscence therapy, and music therapy. The scores of the MMSE and the ADAS significantly improved in the experimental group compared to the control group with the following effect sizes: 0.47 for MMSE and 0.35 for ADAS. Assuming the same effect sizes in a repeated measures ANOVA model (2 groups and 2 repeated assessments), setting the statistical power at 90% and a significance level equal to 0.05, the expected sample size should be 38 to capture an effect on the MMSE and 68 subjects for ADAS. Considering a 1:1 ratio between cases and controls and a potential dropout rate of 15%, the overall sample size should be 78 subjects (39 cases and 39 controls).

It is hypothesized that this sample dimension is more than sufficient to grasp a variation also for secondary outcomes for which a treatment effect size is assumed of a similar or higher entity than that identified for the primary outcome.

### Recruitment

Patients will be selected by the outpatient Center for Cognitive Disorders and Dementia (CDCD) of the Clinical Unit of Neurology of the IRCCS INRCA in the Ancona branch. These patients, their informal caregivers, and his/her support administrator will be contacted to schedule a visit with the psychologist. Once the compliance with the inclusion and exclusion criteria of the study will be verified and informed consent will be obtained, the psychologist will proceed with the baseline evaluation and with the acquisition of gait assessment parameters through the G-Walk sensor. Subsequently, the subject will be randomly assigned to one of the two study groups. The trial will start in June 2022 and is expected to end in January 2023.

### Intervention

**Figure 1:**
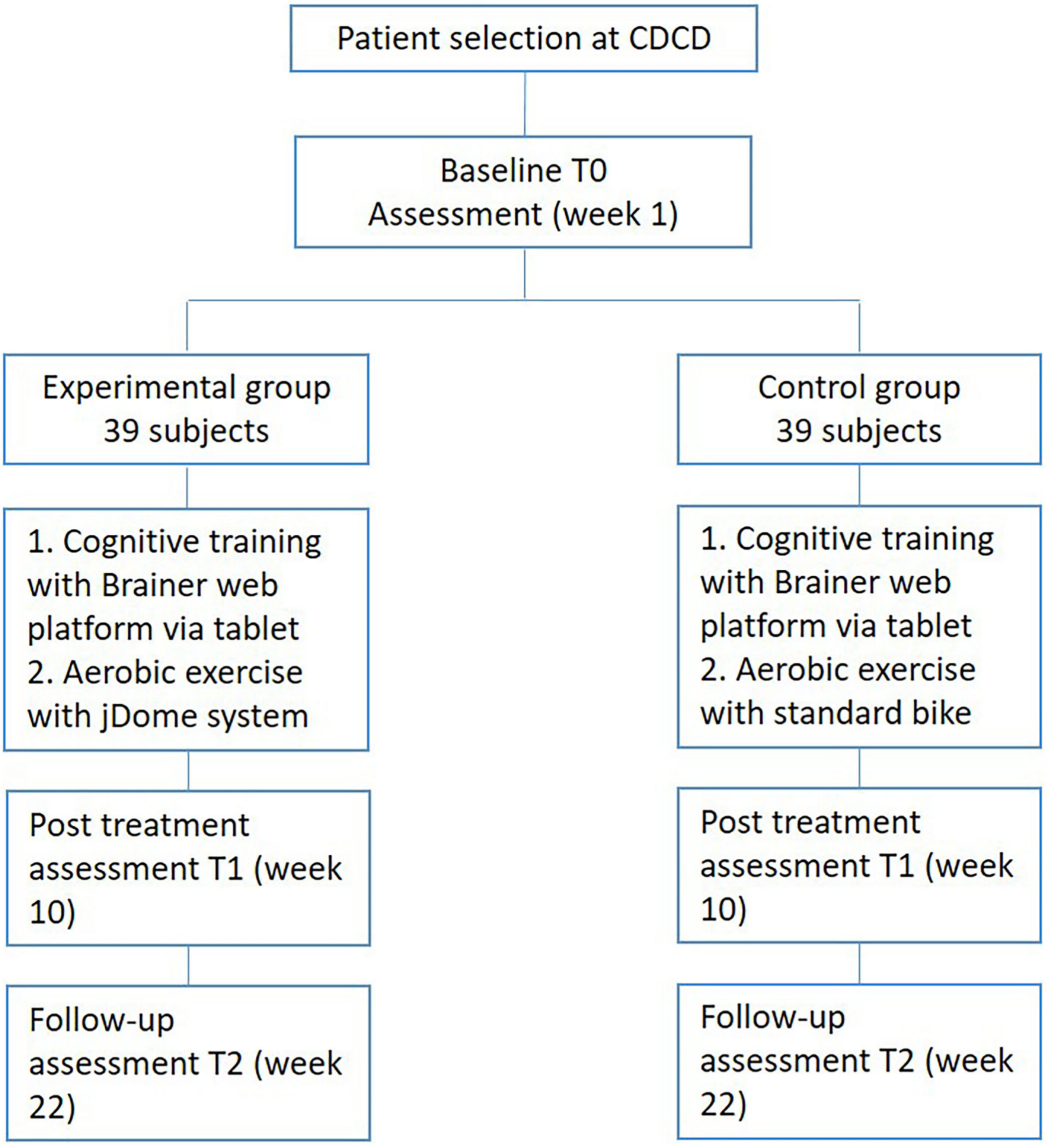
Flowchart of the patient selection.

For this study, 78 patients with mild Alzheimer's dementia will be enrolled. Sixteen treatment sessions of 60 min will be conducted for each group (2 training sessions per week, for 8 weeks), involving 1 patient at a time. Cardiac and respiratory activity monitoring will be conducted during aerobic treatments to detect heart rate and breathing frequency. Figure 1 describes the flowchart of the patient selection.

Each session for the Experimental Group will involve the following activities:

- 30 min of cognitive training with the tablet.
- 30 min of aerobic training with the JDOME system.

Each session for the Control Group will involve the following activities:

- 30 min of cognitive training with a tablet.
- 30 min of aerobic training with a standard exercise bike.

### Computerized cognitive training description (EG and CG)

Each participant will be provided with a touchscreen tablet to access the web platform “Brainer, Professional Brain Trainer” (49). The cognitive training program includes repeated exercises involving memory, attention, language, executive functions, and visual-spatial and perceptual-motor skills. Each session will begin with an exercise asking the participant to draw a line on the screen to join the numbered dots together to help him/her become familiar with the touch screen. Following there will be six exercises, different from session to session, that will stimulate all the target cognitive functions of the training. Each exercise has three levels of difficulty that will be proposed incrementally to all subjects. The scheme of the session is fixed to guarantee everyone the same standardized treatment. The cognitive training sessions are led by the psychologist and the tablet is not expected to be used at home. Computerized cognitive training will be done equally in both study groups.

Each meeting will take place according to this scheme:

- 05 min—space-time orientation.
- 20 min—cognitive exercises carried out with the “Brainer” web platform
- 05 min—conclusion and greetings.

During the first four sessions and the sessions from 13 to 16. the exercises will be organized as follows:

- Familiarization with the touch screen (1 exercise)
- Attention (1 exercise)
- Executive functions (1 exercise)
- Learning and memory (1 exercise)
- Language (2 exercises)
- Perceptual-motor skills (1 exercise).

During the sessions from 5 to 8, the exercises will be organized as follows:

- Familiarization with the touch screen (1 exercise)
- perceptual-motor skills (1 exercise)
- Language (2 exercises)
- Learning and memory (1 exercise)
- Executive functions (1 exercise)
- Attention (1 exercise).

During the sessions from 9 to 12, the exercises will be organized as follows:

- Familiarization with the touch screen (1 exercise)
- Learning and memory (1 exercise)
- Executive functions (1 exercise)
- Attention (1 exercise)
- perceptual-motor skills (1 exercise)
- Language (2 exercises).

#### Brainer, professional brain trainer web platform description

Brainer is a class I medical device with a relative CE mark clinically tested for the cognitive rehabilitation of people with mild stage dementia. It is accessed through the Brainer web platform, www.brainer.it, and can therefore be used on any PC or tablet with an internet connection. The macro section “exercise profiles” offer the possibility to define a sequence of exercises to be assigned to a specific subject defining a personalized rehabilitation on the basis of the deficits of the participant and their severity. The platform contains 78 exercises in the five cognitive areas involved in the degeneration process of dementia (complex attention, executive functions, learning and memory, language, and perceptual-motor). The exercises have 3 levels of increasing difficulty, and each level requires an average of 5 attempts to move on to the next.

### Moderate-intensity aerobic training (EG and CG)

The physical component of training with the standard exercise bike or with the jDome system consists in carrying out the aerobic activity of moderate intensity supervised by the physiotherapist, as well as by the psychologist. Aerobic activity is structured by adapting guidelines from the American College of Sports Medicine (ACSM) Guidelines for Exercise Testing and Prescription (54) and indications provided by similar studies in the literature (33, 36). The duration of 25 min of training plus 5 min of cool down was selected both as an appropriate length to encourage adequate commitment and involvement of the participants in the exercise, and because a duration between 20 and 30 min is defined in the literature as a duration necessary to adequately increase heart rate and oxygen consumption and therefore suitable to significantly affect the disease (55). To define the intensity of aerobic exercise, the calculation of the heart rate using the pulse oximeter will be used. According to the literature, physical activity can be defined as moderate when it induces an effort in the subject to cause an increase in heart rate by 40–60% of his maximum heart rate (55–57). The formula widely used in literature will be used to calculate heart rate HRmax = 220-age in years (36). According to the literature recommendations (36), the physical training will be individualized and calibrated on the tolerance and physical response of the individual participant, but each session will aim to reach a target heart rate and maintain it for an increasing period of time during the sessions (Table 1). In the event that the participant fails to reach the target heart rate for that session, he/she will continue pedaling at the maximum speed and resistance level that he/she can maintain in that session without over-fatigue. As recommended by other similar studies in the literature (33, 36), the participant will be constantly monitored during the activity to keep the training at a moderate intensity level to avoid determining too much effort, more typical of a high training intensity or a minimal effort of a light intensity activity, which is not sufficient to bring about a change.

**Table 1:** Intensity and duration of aerobic exercise session by session.

**Session**	**Intensity prescribed**	**Minimum duration of**
	**(% of maximum**	**the maintenance period**
	**heart rate)**	**of the prescribed intensity**
1–4	55–60	10
5–6	55–60	20
7–8	60–65	10–15
9–12	60–65	15–25
13–16	60–65	15–25

According to the literature, the intensity of physical activity that induces health benefits is moderate, which increases the metabolism by 3–6 times compared to the rest of the situation, increases the heart rate, and determines a slight subjective sensation of shortness of breath and warming^1^. Considering this, three indices will be used to monitor the level of intensity of the exercise (36) at the beginning and then every 5 min:

- heart rate not exceeding 60–65% of the HRmax (36, 58),

- breathing difficulties, i.e., the ability to speak coherently without breathing difficulties while pedaling (53),

- signs and symptoms of fatigue (e.g., sweating level, skin color, breathing patterns, and abnormal behavior) (36).

During the first four training sessions (sessions 1–4), the subject will be asked to begin pedaling at a low number of revolutions per minute (RPM), one revolution every 30–40 s with zero resistance for 2–5 min. RPM and resistance will be increased alternately every 2–5 min until the target heart rate for the session is reached, or earlier if the participant demonstrates that they cannot tolerate a further increase in RPM or resistance, thus reaching the maximum intensity level of that specific session.

The participant will then be encouraged to continue pedaling at the peak level for 10 min tolerated. In the following sessions, the duration of the pedaling at the maximum level of intensity, scheduled or reachable by the subject, will be gradually lengthened up to 20 min. Each new session will see a gradual increase in intensity and the time spent in the new intensity.

From session seven, the physiotherapist will attempt to increase the intensity by 60–65% of their maximum heart rate or until the participant's tolerance is reached. The participant will cycle at this new level for 10–15 min as tolerated. The duration of the pedaling will be gradually extended in subsequent sessions until the participant can pedal continuously for 25 min at this new maximum intensity level.

At the end of each pedaling session, the participant will be asked to cool down by gradually decreasing the RPM and resistance to zero over 5 min.

#### Description of the jDome system for aerobic training (EG)

**Figure 2:**
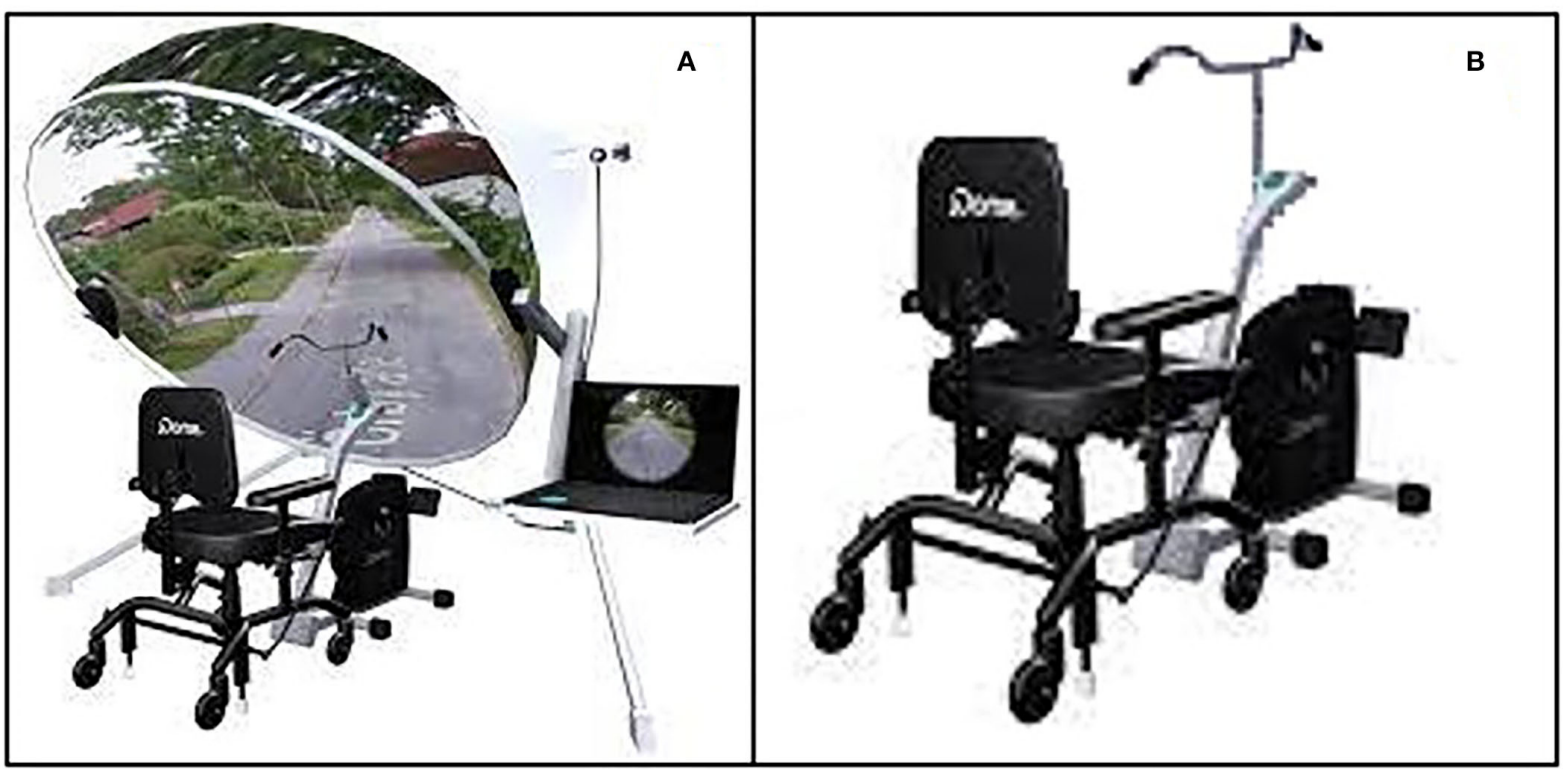
**(A)** Exercise bike associated with the JDome virtual reality system. **(B)** Standard exercise bike obtained from the JDome System.

To carry out the physical component of the training, the subjects of the experimental group will use the jDome system (Figure 2A). jDome system connects a static bike to a monitor in which the path along which the subject is pedaling is projected, thanks to Google Street View. The projection of the path on the monitor will allow the psychologist to carry out a generalized stimulation of cognitive functions and to recall the memories associated with paths and places known to the subject. At the same time, the recall of emotionally significant memories related to the places found along the way could support the motivation for physical activity.

Physical training sessions with the jDome system will follow the following pattern:

- 05 min ✓ introduction, promotion of motivation to participate, and space-time orientation activities;
- 20 min ✓ pedaling controlled with an oximeter in the streets of one of the places indicated as emotionally significant by the subject in the initial interview. Recovery of memories associated with the places identified. Re-enactment of the emotions associated with them. Generalized stimulation of other cognitive functions (attention, language, visual-spatial skills, and spatial orientation).
- 05 min ✓ cool-down, collection of impressions and synthesis of what has been experienced, and appointment for the next session.

#### Standard exercise bike for physical training (CG)

The participant of the control group will use a standard static bike (Figure 2B) and will be motivated by the psychologist to persist in the aerobic effort to achieve the set goal. The exercise bike used will be the one that makes up the jDome system but, in this case, the jDome monitor for virtual reality will be removed or disconnected. This choice makes it possible to avoid introducing confounding variables in the experimentation related to the possible use of a different exercise bike for the physical training of this group.

Physical training sessions with standard exercise bikes will be structured as follows:

- 05 min → introduction, promotion of motivation to participate, and space-time orientation activities;
- 20 min → controlled pedaling with an oximeter, interventions by the psychologist aimed at maintaining motivation to exercise;
- 05 min → cool-down, collection of impressions, and appointment for the next session.

### Outcomes

All outcomes will be measured following a standardized operating procedure. Table 2 shows the primary outcome and the secondary outcomes. All measures are adapted and are valid for this population in Italy.

**Table 2:** Outcomes and clinical assessments.

**Outcome(s)**	**Clinical assessment**
Primary: Cognitive functioning	MMSE and ADAS-cog
Secondary: Mood	CSDD
Secondary: Psychological and behavioral disorders	NPI
Secondary: Physical performance	SPPB and 6MWT though G-Walk sensor
Secondary: Quality of life	QoL-AD

*MSE, Mini Mental State Examination; ADAS-Cog, Cognitive subscale of the Alzheimer's Disease Assessment Scale; CSDD, Cornell Scale for Depression in dementia; NPI, Neuropsychiatric Inventory; SPPB, Short Physical Performance Battery; 6MWT, 6 Minute Walking Test; QoL-AD, Quality of Life-Alzheimer's Disease scale*.

A summary of all data collected and when they were collected is provided in Table 3.

**Table 3:** Schedule of assessment and outcome measures.

	**R**	**T0**	**T1**	**T2**
Socio-demographic checklist	✓			
Semi-structured interview about the previous level of physical activity and habit of cycling over the course of life	✓			
Functional Ambulation Category (FAC)	✓			
Tinetti's Scale or Performance-Oriented Mobility Assessment (POMA)	✓			
Mini Mental State Examination (MMSE)		✓	✓	✓
Alzheimer's Disease Assessment Scale- Cognitive subscale (ADAS-cog)		✓	✓	✓
Cornell Scale for Depression in dementia (CSDD)		✓	✓	✓
Neuropsychiatric Inventory (NPI)		✓	✓	✓
Short Physical Performance Battery (SPPB)		✓	✓	✓
6 Minute walking test (6MWT)		✓	✓	✓
Quality of Life - Alzheimer's Disease scale (QoL-AD)		✓	✓	✓
“Treatment monitoring” and “virtual cycling experience evaluation” sheets	Before and after each session			
“Monitoring of the cycling experience” sheet and:	During each aerobic session: at the beginning and every 5 min			
- Pulse oximeter for heart rate meter			
- Talk test to monitor breathing difficulties			
- Observation of signs and symptoms of fatigue			

*R, Recruitment; T0, Start of treatment; T1, end of treatment; T2, follow-up (3 months after the end of treatment)*.

The scales which will be used during the evaluations are described below.

#### Ad hoc semi-structured interview about the previous level of physical activity and habit of cycling over the course of life

The scale consists of 7 open-ended questions.

#### Functional ambulation categories

The scale is used to classify the severity level of gait disturbances in neurological disorders. It provides a hierarchical classification from level 0 (impossible walking) to level 5 (no limitation) (59–61).

#### Tinetti's scale or performance-oriented mobility assessment

Tinetti scale is a tool used to evaluate balance and gait performance. The test is clinically used to determine the mobility status of a subject or to assess changes in balance and gait time. The total POMA (POMA-T) consists of two sub-scales: the balance evaluation scale (“balance scale” or POMA-B) and the gait evaluation scale (“gait scale” or POMA-G). Scores equal to or <1 indicate a non-ambulatory subject, scores between 2 and 19 indicate a walking subject at risk of falling, scores equal to or greater than 20 indicate a walker with a low risk of falling (62).

#### Mini-mental state examination

Mini-Mental State Examination was designed as a clinical method for grading cognitive impairment. The score ranges from 0 to 30: scores ≥24 indicate normality, between 18 and 23 indicate mild cognitive impairment, between 11 and 17 indicate moderate cognitive deficits, and scores ≤ 10 severe cognitive impairments. The reported score is corrected according to age and education (63).

#### Alzheimer's disease assessment scale

It is considered the “gold standard” in dementia treatment evaluation studies with a good size of treatment effect and good sensitivity to change (29). The questionnaire consists of 11 items that investigate: short and medium-term memory, language, praxia (simple, constructive, ideational), and temporal–spatial orientation. The score ranges from 0 (no cognitive impairment) to 70 (maximum cognitive impairment) (64–66).

#### Cornell scale for depression in dementia

The scale contains 19 items evaluating signs and symptoms of major depression in individuals with dementia (67). Each item is rated for severity on a scale from 0 (absent) to 2 (severe). The information is collected through clinical observation and two semi-structured interviews, one addressed to the caregiver and one to the person with dementia. The total score, given by the sum of the 19 items, if lower than 6 indicates the absence of depressive symptoms, if higher than 10 it indicates a probable major depression and finally if higher than 18 it indicates the presence of major depression.

#### Neuropsychiatric inventory

The scale evaluates 12 neuropsychiatric disturbances common in dementia, namely delusions, hallucinations, agitation, dysphoria, anxiety, apathy, irritability, euphoria, disinhibition, aberrant motor behavior, night-time behavior disturbances, and appetite and eating abnormalities. A total NPI score and a total caregiver distress score are calculated. The total NPI score, which ranges from 1 to 144, is the product of the frequency of manifestation and the severity of each disorder. Higher scores indicate more frequent and more severe behavioral problems (68).

#### Short physical performance battery

The SPPB scale is a short battery of tests designed to assess the function of the lower limbs. This scale consists of 3 different sections: balance assessment, evaluation of walking for 4 linear meters, evaluation of the ability to perform for 5 consecutive times, and the sit to stand from a chair without using the upper limbs. The total scale score, therefore, has a range from 0 to 12. A total score below 10 indicates frailty and a high risk of disability and falls. The 1-point change in score from pre- to post-test is of clinical relevance (69–71).

#### The 6-minute walking test and G-sensor

6MWT is a test that allows the measurement of a patient's residual functional capacity. The test is performed by asking the patient to walk for 6 min along a corridor with a rigid walking surface. The 6MWT is based on a so-called self-paced mode, i.e., the patient chooses the intensity of effort. The minimum detectable change at the 95% confidence level (MDC95) is 28.1 m in frail elderly people with dementia (72, 73).

The gait analysis through the use of the *G-Sensor* during the execution of the 6MWT will also be carried out in participants. The G-sensor is a wearable system for gait and movement assessment. It consists of 4 inertial sensors and a GPS. It must be positioned through a band at the level of the pelvis. The system guarantees an operating autonomy of 8 h and an unlimited range of action, thanks to the internal memory. It allows to have a kinematic evaluation of the trunk and the space–time parameters recorded during the various integrated tests and protocols.

#### Quality of life—Alzheimer's disease scale

The scale includes 13 items that evaluate subjective (e.g., perceived quality of life and psychological wellbeing) and objective (e.g., behavioral competence and environment) components of the quality of life. Items are rated by subjects with dementia on a 4-point scale from 1 (poor) to 4 (excellent). Higher scores indicate a better quality of life (74).

*Ad hoc sheet on “Treatment monitoring”* includes 5 items to be assessed on a 4-point scale:

- 1 item to be administered before the session and concerns the level of interest and motivation to participate;
- 3 items to be administered at the beginning and after the session about the level of verbal communication, mood, and the level of agitation;
- 1 item to be administered only after the session about the fun level. Answers can be provided on a 4-point scale from the worst condition (e.g., no interest) to the best condition (much interest).

*Ad hoc sheet on “Evaluation of the virtual cycling experience”* includes 9 items, the domains of which are:

- Participant's mood at the end of the virtual bike tour. The response scale is a visual-analog scale with five faces like colorful emoticons that express from great happiness to deep sadness or unhappiness. The participant only has to indicate which face best identifies their mood. The scale is administered by saying: “This face shows happiness, this face appears a little less happy, this other one still a little less (moving the finger along the scale from left to right), to the point of extreme sadness. Take a look at these faces and choose the one that shows how you feel right now.” The visual analog scale has proved useful and sensitive to the needs of subjects with dementia (38, 71).
- The level of satisfaction of the virtual cycling experience was adapted from D'Cunha et al. (38). These 6 items are open-ended questions.
- The place where the cycling activity of the specific session took place and whether this was chosen by the person or not. These two items are filled in by the psychologist.

*Ad hoc sheet on “Monitoring of the cycling experience”* includes 3 monitoring indices of the intensity of physical activity concerning:

- Heart rate measurement by the oximeter. The goal is to keep it within the values that correspond to the maximum heart rate percentage (% HRmax) target of the session (Table 1) or in any case not higher than 60–65% of HRmax. Maximum heart rate is estimated using a standard equation HRmax = 220—age, although significant variability is associated with this estimate (SD, ± 10 to 12 beats per minute). Indeed, heart rate can be affected by a number of factors other than the intensity of the current exercise, such as environmental conditions that affect heat dissipation (in particular ambient heat, convective airflow, and humidity), degree of rest or overtraining of the individual, stress, heart disease, and medications (36, 58). Other indices will also be used for this.
- Talk testing (TT) compares the way of speaking that the subject has in the resting phase with what he/she has when he/she reaches the maximum expected exercise intensity. If during the exercise the subject speaks with breathlessness, the intensity of the exercise is above his ventilatory threshold typical of moderate intensity. The goal of the use of TT, therefore, is to keep the intensity of the exercise within a range in which breathlessness is absent, but there is minimal difficulty with the subjective sensation of mild shortness of breath (58, 75).
- Observation of signs and symptoms (e.g., sweating level, skin color, breathing patterns, and abnormal behaviors) during the exercise. In fact, at a moderate intensity level, the participant should appear warm, but not sweating, or short of breath (36, 75).

### Risk management and mitigation

Participation in the study involves minimal risks concerning first of all the use of the jDome system and in particular the possibility of developing sensations of visual fatigue, dizziness, and nausea from exposure to the jDome system monitor (49, 52). Other minimal risks could relate to the use of the exercise bike, in particular the risk of falling or physical fatigue (38, 55). To reduce the risk of musculoskeletal injuries, a moderate start of physical activity is provided with gradual progress toward higher levels of intensity; to limit fatigue, heart rate and the ability to speak fluently during the sessions will be constantly monitored, evaluating any breathing difficulties. In general, stationary bikes appear particularly safe for seniors with Alzheimer's because they stop if the person stops pedaling, thus reducing the risk of falls. To reduce the risk of skidding or falling due to dizziness after using the jDome system, recovery time in a sitting position will be guaranteed without being exposed to a monitor. During this phase, impressions will be collected.

In the event that a participant starts showing one or more of these signs or symptoms (e.g., dizziness, fatigue, and back pain), participation will be immediately stopped. If any element/behavior dangerous to the physical and/or mental health of the participant is noticed, the psychologist who will follow each session will expose the subject and his / her caregiver to the problems encountered and will eventually interrupt participation in the study. Overall, the benefits of being physically active outweigh the risks in all age groups.

### Data management

The procedures reported in the study regarding the conduct, development, and documentation have been prepared to ensure compliance with the ethical principles set out in the Declaration of Helsinki and its revisions. The research design also took into account the directives provided by the General Data Protection Regulation (GDPR, Regulation (EU) 2016/679) (76). The study will be conducted by taking into account the regulatory requirements and the legal obligations and will be launched following the assessment and approval of the study by an independent Ethics Committee and the completion of the administrative obligations required by the institution where the study is carried out. Furthermore:

- before enrollment, all participants must receive complete information on the study.
- to be enrolled, it will be necessary that the participants or their legal guardians give consent to the processing of personal data in anonymous and aggregate form, under the General Data Protection Regulation (GDPR 2018, Regulation (EU) 2016/679) on the protection of individuals and respect for the processing of personal data (76).
- the participant must be informed that his data can be reviewed by authorized personnel or by members of the relevant ethics committee and by officials of the competent regulatory authorities.
- the participant is also informed and asked to provide informed consent for data retention for up to 15 years from the conclusion of the study.

The processing of personal data and information will take place in compliance with current privacy legislation. All data will be digitized, made anonymous, and archived on a centralized and secure IT platform. They will be marked with a numerical code and will not contain the name of the participant in the study or data that can identify him. The data will be archived in databases of the IRCCS INRCA of Ancona and will be kept for 15 years beyond the end of the study.

### Data analysis

An analysis plan will be defined on the basis of which the analyzes themselves will then be conducted. Data entry will be carried out using special software, providing blocks and data entry checks to reduce the number of entry errors. The quality of the data and their internal consistency will be evaluated using Cronbach's Alpha and other specific tests. The questionnaires will first be checked manually to check the completeness of the compilation and any obvious inconsistencies. Automated routines will then be used to detect outliers and dubious records. In such cases, the necessary data cleaning will be carried out.

The first step of the analysis will be exploratory. The descriptive analysis of the sample will be conducted through the classic techniques of uni- and bi-variate statistical analysis. The categorical variables will be summarized by reporting the absolute and relative frequencies, while for the continuous variables the mean and standard deviation or median and interquartile deviation will be reported depending on the distribution (evaluated through the Shapiro–Wilk test). Significant differences between outcomes and exposures will be compared using the Chi-square test or the Fisher's exact (in the case of categorical variables) or the *T*-test or the Mann-Whitney test (in the case of comparisons of continuous variables between groups depending on the distribution of the same). To check for any distortions due to non-responses during the survey, the characteristics of the subjects in the sample will be compared with those of the non-respondents.

In a second step, the follow-up data analysis will be conducted to evaluate the effectiveness of the J-DOME intervention. This phase of analysis will involve the use of multivariate statistics, in particular, the variance analysis models for repeated measures to identify the factors associated with the variation of the main secondary clinical end-points.

## Discussion

The aim of the present study is to evaluate a treatment protocol for patients with mild Alzheimer's disease aimed at improving firstly cognitive performance, and secondly mood, quality of life, and physical performance. The treatment in question involves two different moments, first, the cognitive training performed using a Brainer platform and second, the aerobic physical activity performed using an exercise bike. Finally, the treatment also uses virtual reality to offer the person a reminiscence activity *via* the jDome system by having him or her ride a bicycle along roads known and important to him or her.

We focus on patients with mild Alzheimer's disease to test whether exercise combined with cognitive training and reminiscence therapy can actually lead to benefits in terms of increased cognitive plasticity and hippocampal neurogenesis. To study the effectiveness of the treatment, we will divide the population into an experimental group, treated with cognitive therapy and physical activity with the jDome system, and a control group, treated instead with cognitive therapy and physical activity with a simple exercise bike. During the virtual course, the person's memories will be stimulated so as to increase motivation for the activity and use and thus train the cognitive functions. With the intervention of a psychologist during the session, cognitive stimulation will also be expanded to other functions, such as attention, language, visuospatial skills, and spatial orientation.

Another important aspect of the protocol is the check of the results obtained not only at the end of the treatment but also after 3 months from the end of the treatment to verify the maintenance and generalization of the results gained.

## Ethics and dissemination

### Ethics and confidentiality

The study was approved by the Ethics Committee of the Istituto Nazionale Ricovero e Cura per Anziani (IRCCS INRCA) on the 31 March 2022. It was recorded in ClinicalTrials.gov on 2 June 2022 with the number NCT05402423. Any protocol modifications will be notified to the above-mentioned Ethics Committee. The same committee is in charge of data monitoring and periodically assesses the progress of the protocol and compliance with what was declared. The principles of the Declaration of Helsinki and Good Clinical Practice guidelines will be adhered to. Participants in this study will provide written informed consent.

Personal data collected during the trial will be handled and stored in accordance with the General Data Protection Regulation (GDPR) 2018. The use of the study data will be controlled by the principal investigator. All data and documentation related to the trial will be stored in accordance with applicable regulatory requirements, and access to data will be restricted to authorized trial personnel.

The acquisition of informed consent from people with Alzheimer's disease is a long-debated issue.

Although degenerative neurological syndromes, including Alzheimer's disease, over time, lead to a progressive decline in cognitive functions and with them the ability to express valid consent, the diagnosis of Alzheimer's disease does not in itself lead to the loss of this ability. The legal capacity and the capacity to act remain, unless proven otherwise, from the age of majority until the death of the person. Only a judicial measure can protect the person with dementia who is unable to provide informed consent by appointing a legal representative.

A person with dementia can maintain decision-making skills about some or many aspects of their life and health for a long time.

From a neuropsychological point of view, the impairment of executive functions (abstraction ability, problem-solving, judgment and criticism, planning, planning, farsightedness, “decision making”…) is directly proportional to the decrease in capacity both in a general sense in various fields, such as health and economic decisions, and driving skills. However, these cognitive functions can be spared in the early stages of the disorder (MMSE > 19) unlike others that are affected early, such as memory and orientation.

It is also a common clinical experience that, even if unable to understand the contents of a standard “informed consent” form (which must certainly be simplified), a person with dementia is often able to express his/her choices in line with his lifestyle, preferences, and values. This underlines the importance of preserving the possibility for potential participants to use their skills to share possible choices.

Informed consent is a legal condition in which a person accepts an action that is proposed to him/her (in our case, active participation in a clinical trial). To be “informed” the consent must be based on a full understanding of the action itself and the implications it can bring. This implies that:

- every effort must be made to guarantee and respect any residual capacity for autonomous decision, considering consent as an instrument through which the subject realizes his autonomy.
- the autonomy of the subject requires that all information be understood
- the person's consent presupposes his/her ability to choose freely on the basis of his preferences, moral values, life stages, and circumstances.

It is therefore necessary first of all to inform the person with dementia, adapting the information to the cognitive abilities of the same, making every effort so that the patient can directly or indirectly communicate his preferences. With this in mind, the opinion of family members, for example, may be requested but considered secondary to that of the patient.

The person with dementia who gave his informed consent to participate and is not comfortable during the sessions may at any time withdraw from the trial without any consequences.

In the specific case of our clinical trial, neither serious harmful effects on the person with dementia are foreseeable nor is there bad faith in our treatment proposal. On the contrary, literature studies demonstrate the potential benefits of the proposed intervention.

With this in mind, we proceed to ask the person with mild stage dementia to provide their informed consent to participate, and we strive to:

- ensure that he/she clearly understands the content of the information sheet and the consequences of his/her participation;

-create the best conditions in which he/her can ask questions and express his/her will;

- monitor throughout the course of the trial the persistence of his/her willingness to participate.

#### Procedure for requesting informed consent

During the clinical interview, the contextual assessment of the ability to express an autonomous choice will be carried out, assessing the presence of:

- Ability to express a choice;
- Ability to understand information relating to consent;
- Ability to give due weight to the situation and its possible consequences;
- Ability to use information rationally.

In the event that this evaluation gives a positive result, informed consent will be acquired from the person himself. Time and effort will be devoted to providing correct and full information, the information sheets, and the consent form will be read together with the person with dementia and their caregiver, the opportunity to ask questions will be given, and the best conditions will be created to make a decision. An additional opinion will be requested from the main and reference caregiver on whether or not the person with Alzheimer's should participate in the project, what if his/her wishes and feelings about participation may not have been expressed. If the subject then shows signs of dissent before and during each training session or shows behaviors that suggest that he/she is no longer willing to participate, the sessions will be terminated and the consent will be automatically withdrawn.

For people unable to express valid consent, on the other hand, the Legislative Decree of 24 June 2003 n. 211 (77) and European Regulation 536/2014 (78) identify in the figure the legally designated representative (support administrator) the person who must be involved in the information process and from whom informed consent must be obtained. Even in this case, however, the will of the person directly concerned to participate in the “double consent” mode will be tested.

### Dissemination of research findings

The study findings will be used for publication in peer-reviewed scientific journals and presentations in scientific meetings. Summaries of the results will also be made available to investigators for dissemination within their clinics.

## Author contributions

Study concept and design: EG. Acquisition of data: EG and MB. Analysis and interpretation of data: RB, EM, and AM. Drafting of the manuscript: EG, EM, RB, and AM. Critical revision of the manuscript for important intellectual content: GP, GR, PC, and LR. Writing—review and editing: EM, RB, SP, and PD'A. All authors have read and agreed to the published version of the manuscript.

## Funding

This work was conducted inside the project INNovazione sociale e tecnologica per le FAMIGLIE che assistono malati affetti da Alzheimer (INNfamiglia) funded by Fondazione Cariverona, Bando Welfare & Famiglia, 2017.

## Conflict of interest

The authors declare that the research was conducted in the absence of any commercial or financial relationships that could be construed as a potential conflict of interest.

## Publisher's note

All claims expressed in this article are solely those of the authors and do not necessarily represent those of their affiliated organizations, or those of the publisher, the editors and the reviewers. Any product that may be evaluated in this article, or claim that may be made by its manufacturer, is not guaranteed or endorsed by the publisher.

## Footnotes

^1^Available online at: https://www.epicentro.iss.it/attivita_fisica/livelli-consigliati.
